# Visualization of the recurrent laryngeal nerve alone versus intraoperative nerve monitoring in primary thyroidectomy: a framework approach to a missing typology

**DOI:** 10.3389/fsurg.2023.1176511

**Published:** 2023-07-24

**Authors:** Dimitris Papagoras, Georgios Tzikos, Gerasimos Douridas, Polyvios Arseniou, Dimitrios Panagiotou, Maria Kanara, Theodosios Papavramidis

**Affiliations:** ^1^ Department of Surgery, General Hospital of Trikala, Trikala, Greece; ^2^ 1st Propedeutic Department of Surgery, Aristotle University of Thessaloniki, AHEPA University Hospital, Thessaloniki, Greece; ^3^ Department of Surgery, Thriassio General Hospital of Elefsina, Elefsina, Greece

**Keywords:** recurrent laryngeal nerve, visualization, nerve monitoring, thyroidectomy, surgical technique, typology, interventional bias

## Abstract

**Introduction:**

Surgical studies evaluating a device or technology in comparison to an established surgical technique should accurately report all the important components of the surgical technique in order to reduce the risk of intervention bias. In the debate of visualization of the recurrent laryngeal nerve alone (VONA) versus intraoperative nerve monitoring (IONM) during thyroidectomy, surgical technique plays a key role in both strategies. Our aim was to investigate whether the surgical technique was considered as a risk of intervention bias by relevant meta-analyses and reviews and if steps of surgical intervention were described in their included studies.

**Methods:**

We searched PUBMED, CENTRAL—Cochrane library, PROSPERO and GOOGLE for reviews and meta-analyses focusing on the comparison of IONM to VONA in primary open thyroidectomy. Τhen, primary studies were extracted from their reference lists. We developed a typology for surgical technique applied in primary studies and a framework approach for the evaluation of this typology by the meta-analyses and reviews.

**Results:**

Twelve meta-analyses, one review (388,252 nerves at risk), and 84 primary studies (128,720 patients) were included. Five meta-analyses considered the absence of typology regarding the surgical technique as a source of intervention bias; 48 primary studies (57.14%) provided information about at least one item of the typology components and only 1 for all of them.

**Discussion:**

Surgical technique of thyroidectomy in terms of a typology is underreported in studies and undervalued by meta-analyses comparing VONA to IONM. This missing typology should be reconsidered in the comparative evaluation of these two strategies.

## 1. Introduction

Surgical trials represent scientific efforts to answer outcome-focused questions, pertinent to surgical interventions (1). Randomized controlled trials provide the highest level of evidence when two or more interventions need to be evaluated (2). And when it comes to interventions, a critical parameter which obviously needs to be evaluated is the interventional bias, which refers to the biases that result from systematic differences in the way in which the intervention was carried out between groups. This level of evidence, especially the interventional bias assessment, seems not to be reached efficiently even in recently published meta-analytical studies comparing the use of intraoperative nerve monitoring (IONM) versus visualization of the recurrent laryngeal nerve alone (VONA) in primary thyroidectomy, in regard of vocal cord palsy (VCP) prevention (3). Additionally, the available results of meta-analyses and systematic reviews related to the subject are conflicting and inconclusive as well (4).

It has also been acknowledged that in the majority of published surgical trials little attention has been paid to standardization of the evaluated intervention(s) (5). Detailed definition, accurate description of surgical technique, potential variability in performance by participating surgeons and quality control of the intervention under evaluation are scarcely stated clearly in surgical trials (6). This methodological prerequisite of standardizing and reporting the surgical technique in trials is defined as typology, a term elaborated by Blencowe et al. (6) in response to the undeniable fact that “the reporting of surgical intervention is in need of immediate improvement” (7). The absence of standardization of the surgical technique(s) produces heterogeneity in the context of qualitative analysis of methodology (8, 9) and represents a clear risk of intervention bias, questioning the internal and external validity of the study (10). Furthermore, the combined analysis of several studies (i.e., meta-analyses, reviews) presenting with the same methodological oversight (11), inevitably leads to inconclusive accumulation of heterogeneous data that are clinically meaningless (12, 13). This (missing) typology in surgical research underlines the determinative importance of demarcating an intellectual framework for standardization of surgical procedures (14), which would allow the results of a study, regarding the efficiency and accuracy of the reported surgical intervention, to be both reproducible and comparable. The surgical intervention under investigation must be “dissected” on those “active ingredients”, the structured performance of which is substantial for the optimal outcome (6). These surgical steps, described meticulously, compose an invaluable mindset (15), a virtual “visualization” of the procedure, which should be easily and safely reproduced in everyday practice (10).

When comparing IONM versus VONA, the surgical technique represents the keystone element of the outcome in question, i.e., prevention of VCP (16). Indeed, regardless of the use of any type of IONM, the surgical technique and, in particular, the sound dissection of the recurrent laryngeal nerve (RLN) represents the condicio sine qua non in thyroidectomy (17, 18). Therefore, it would be anticipated that surgical trials provide a precise definition of the technique and a detailed description of all their subcomponents by which the quoted outcome is achieved. Moreover, meta-analyses and reviews should consider if the surgical technique is clearly reported in included studies (19). Instead, to our knowledge, there are only two reviews (4, 20) assessing qualitatively the meta-analyses, which address the role of IONM versus VONA in thyroidectomy. It is of interest that although both of the beforementioned studies denoted an overall poor reporting and methodological quality, none of them considered the absence of a standardized surgical technique as a major risk of intervention bias. Furthermore, two recently published meta-analyses focusing exclusively in the evaluation of a variety of aspects of IONM, totally detach the use of this technology from the surgical technique (21, 22).

Thus, the aim of this review was to perform a qualitative and quantitative analysis focusing on the potential interventional bias regarding the lack of reported standardization of thyroidectomy in meta-analyses or systematic reviews and by extension in all the included by them primary studies comparing VONA versus IONM.

## 2. Materials and methods

First, we decided to arbitrarily describe a typology for the surgical technique regarding RLN visualization during thyroidectomy which should be reported in the primary surgical trials in order the interventional bias to be avoided. Next, we tried to define a framework for the meta-analyses and systematic reviews to assess their approach of this typology. We have adhered to the updated PRISMA 2020 guidelines for reporting reviews (23). Accumulated data pertinent to technical issues of thyroid surgery allow us not only to evaluate the reporting quality of the studies but also to formulate knowledge reflecting the “state of things” with reference to thyroidectomy technique in surgical literature.

### 2.1. Eligibility criteria

We included all the meta-analyses and systematic reviews focusing on VONA versus IONM in primary thyroidectomy regarding VCP. We excluded meta-analyses, reviews and primary clinical studies: (a) focusing on non-conventional surgical techniques such as endoscopic, minimally invasive or robotic thyroidectomy, as they represent a totally different approach of thyroidectomy and dissimilar handling of RLN to the open technique, (b) dealing with reoperations of the thyroid, because they resist, in contrast to primary surgery, to standardization due to the uncountable variety of the postoperative loco-regional conditions, and (c) providing exclusively an analysis of specific aspects of any type of IONM without comparing them with VONA, obviating the obligation to report technical details regarding the dissection of RLN. After accumulating the eligible studies based on the beforementioned inclusion and exclusion criteria, we extracted all the primary clinical studies from their reference list which were included in a quantitative and qualitative analysis of reported standardization of surgical technique focusing on RLN safeguarding and VCP.

### 2.2. Search strategy

During November 2022 (last access: 20/11/2022), we browsed in PUBMED (Central/MEDLINE), CENTRAL (Cochrane library for meta-analyses and reviews), PROSPERO (registered meta-analyses, reviews or clinical trials) and GOOGLE search engine without any language or time restrictions. Key words and search terms were: #1 thyroidectomy or thyroid surgery and surgical technique, #2 dissection or visual identification and recurrent laryngeal nerve or inferior laryngeal nerve, #3 intraoperative nerve monitoring or nerve stimulation or IONM and #4 meta-analysis or systematic review. The first two authors were responsible for screening the retrieved studies independently, after careful reading of the Title and Abstract, and finally triaging material according to predetermined selection criteria. No automation selection tools were used.

### 2.3. Data collection process

We developed *a priori* (i) a typology (6) for grouping components (items) reflecting important aspects of the surgical technique regarding RLN visualization during thyroidectomy (24–27) that should have been reported in the primary studies (trials) extracted from the meta-analyses and systematic review included in our analysis and (ii) a framework to investigate if these meta-analyses and systematic reviews evaluate the report of this typology in their primary studies. According to the framework, we searched in the meta-analyses/systematic reviews for the following two items: (i) specifically reported search terms such as “surgical technique” or “identification/dissection technique/type/level of visualization” of RLN, (ii) reported risk of intervention bias.

Concerning the typology of the surgical technique, it consisted of the following three items: (i) initial identification type/level/approach of dissection of the RLN (superior, lateral, and inferior) (24) (ii) extend of RLN exposure (total or partial) (26) (iii) the dissection plan/technique of thyroidectomy (capsular or extracapsular) (17, 25).

Additionally, we expanded our search for finding references in the text supporting these items (28). The first three authors worked independently on the collection of all data derived from the included meta-analyses, reviews and primary studies. Data regarding year of publication, design of the study, publication journal, number and origin of the authors, specialty of the first author, number of the patients, nerves at risk (NaR), and IONM type as reported in the title, and finally, the authors' conflict of interest statement were also retrieved and tabulated.

### 2.4. Primary and secondary outcomes

We set *a priori* as a primary aim to appraise and rate the framework by which the published meta-analyses and systematic reviews investigate the typology of surgical technique and the extent up to which the component studies reflect this typology of thyroid surgery in their methodology. As a secondary aim set, we searched whether the meta-analyses and systematic reviews incorporate in their risk of bias assessment the issue of industry funding (29), in conjunction with the reported conflict of interest of authors (30). Additionally, we searched if the specific type of IONM utilized was also reported in the title of their papers.

### 2.5. Data synthesis

Data regarding the framework approach of meta-analyses and reviews were tabulated in order to display the figures and the percentages specific to the following items: (a) use of search terms “surgical technique” and “identification technique of RLN”, (b) assessment of risk of intervention bias, (c) assessment of risk of industry bias, and (d) the authors' conflict of interest statement.

Data pertinent to the described typology were tabulated both as natural numbers and percentages of the primary studies that reported the following items: (i) the identification technique of RLN, (ii) the extent of RLN exposure (total or partial), (iii) the dissection plane of thyroidectomy categorized as either capsular or extra-capsular, (iv) the cited references supporting items (i), (ii) and (iii) and, lastly, (v) the authors' conflict of interest statement. Furthermore, in cases which the procedure of thyroidectomy was described in detail, we categorized the reported identification technique of RLN in superior, lateral or inferior level/type/approach according to Goldenberg and Randolph (24).

During web or live meetings among all authors any kind of conflicts regarding data collection and interpretation were settled through interactive discussion. Microsoft Excel 2019 was used for data input and processing.

## 3. Results

**Figure 1:**
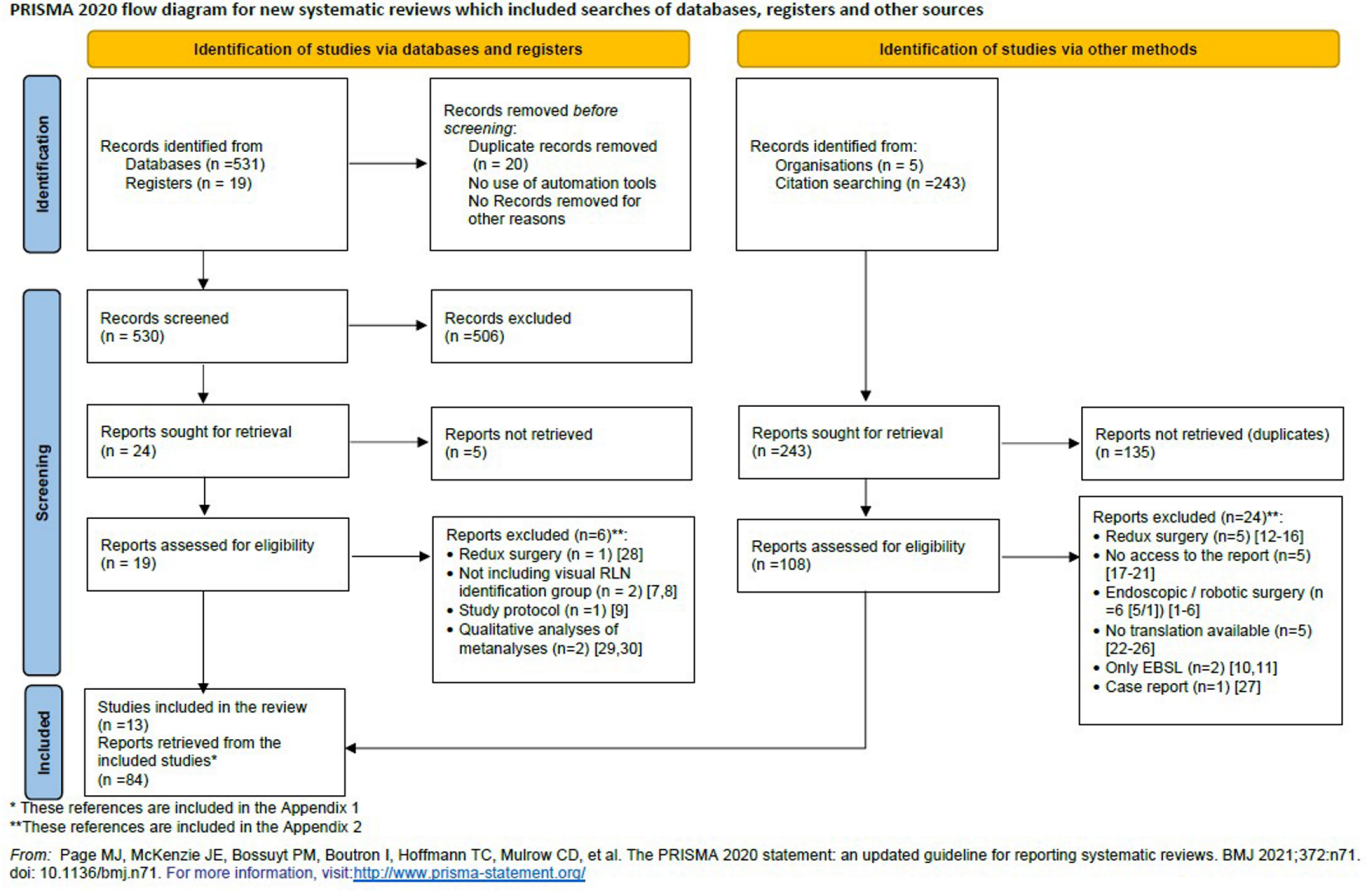
The PRISMA 2020 flow diagram.

Twelve meta-analyses (3, 31–41) and one review (42) met criteria set and were included for data extraction. From these 13 meta-analyses/reviews we extracted 84 primary studies (see reference list in Supplementary Appendix 1). The PRISMA 2020 flow diagram is depicted in Figure 1.

### 3.1. Meta-analyses

Basic characteristics of the meta-analyses/review and studies are depicted in Table 1. The 13 metanalyses/review (3, 31–42) comprised of 243 primary studies including 128,720 patients with 388,252 NaR. The number of patients was omitted in four meta-analyses (23, 35, 39, 41). After careful evaluation of the full text for each article included in our analysis we detected the following discrepancies: (1) erratic reference list in two studies (36, 41), (2) miscalculation of total number of studies in one study (36), (3) inaccurate categorization of studies in one study (39), (4) mismatch between the number of citations and the authors name in the text in one study (41), and (5) inaccurate quotation of results that are opposite to the conclusion of the cited primary study in one meta-analysis (31).

**Table 1:** Basic characteristics of the meta-analyses and the review included in the analysis.

	First author	Number of authors	Specialty^b^	Region	Publication (year of publication)	Number of studies included	Patients	Nerves at risk
1	Davey (3)	4	General Surgery	Ireland	Am J Surg (2022)	8	25,221	4,977
2	Cirocchi (31)	10	General Surgery	Poland, Italy and USA	Cochrane Library (2019)	5	1,558	2,895
3	Bai (32)	2	General Surgery	China	Scientific Reports (2018)	34	n/r	59,380
4	Wong (33)	4	General Surgery	Hong Kong	Int J Surg (2017)	10	n/r	10,615
5	Yang (34)	6	Head & Neck Surgery	China	Int J Surg (2017)	24	9,203	17,203
6	Lombardi (35)	7	General Surgery	Italy	Surgery (2016)	14	n/r	41,743
7	Malik^a^ (42)	2	Stem Cell & Regenerative Biology	USA and Greece	World J Surg (2016)	17	30,926	44,575
8	Pardal-Refoyo (36)	2	Head & Neck Surgery	Spain	Acta Otorrino-laryngol Esp (2016)	40	33,669	61,844
9	Pisanu (37)	5	General Surgery	Italy	J Surg Res (2014)	20	23,512	35,513
10	Rulli (38)	9	Clinical Science & Translational Medicine	Italy and Belgium	Acta Otorhino-laryngol Ital (2014)	8	3,029	5,257
11	Higgins (39)	6	Head & Neck Surgery	USA	Laryngoscope (2013)	43	n/r	64,699
12	Sanabria (40)	10	General Surgery	Colombia, Italy, USA, Israel and Spain	Eur Arch Otorhinolaryngol (2013)	6	1,602	3,064
13	Zheng (41)	5	Head & Neck Surgery	China	J Formos Med Assoc (2013)	14	n/r	36,487

Meta-analyses (*n* = 12).

^a^ Review (*n* = 1).

^b^ The specialty refers to the specialty of the 1st author; n/r, not reported.

### 3.2. Primary studies

In contrast to the meta-analyses/review, several numbers of studies omitted the number of NaR, reporting only the complete number of patients (*n* = 128,720). Thirty-eight studies (45.24%) did not report the RLN injury rate as a primary endpoint. In all studies where IONM was used, the methodology of the applied device was reported in detail. Most studies were retrospective, with RCTs constituting a minority of 7.14% only. Additionally, in more than half of the primary studies (*n* = 48) the authors did not report if there was any conflict of interest. The basic characteristics of the primary studies are presented in Table 2.

**Table 2:** Characteristics of the primary studies retrieved from the selected meta-analyses and review.

*N* = 84			
IONM use	Intermittent	57	(67.86%)
	Continuous	9	(9.52%)
	Not reported	19	(22.62%)
Study design	Retrospective	48	(57.14%)
	Prospective	30	(35.71%)
	Randomized controlled trials	6	(7.14%)
RLN injury as primary aim	Yes	46	(54.76%)
	No	38	(45.24%)
Disclosure	Not stated	48	(57.14%)
	No conflict of interest	25	(29.76%)
	No funding	4	(4.76%)
	No conflict of interest, but funded	1	(1.19%)
	Funded	4	(4.76%)
	Support stated	2	(2.38%)

IONM, intraoperative nerve monitoring; RLN, recurrent laryngeal nerve.

### 3.3. Framework approach

None of the 13 metanalyses/review included in our analysis incorporated in their strategy research the terms “surgical technique” and “identification technique of RLN”. Additionally, less than half of them (*n* = 5) assessed the risk of intervention bias (31, 33, 37, 39, 41) while only 2 out of 13 studies reported the risk of industry bias (31, 41). Furthermore, the majority of the studies (*n* = 9) reported that there was not any authors' conflict of interest (31–34, 36, 37, 39, 41); four studies omitted this information (35, 38, 40, 42). Whereby in one of these studies (38) the affiliation of the 8th Author in line is the department of Statistical medicine of Medtronic the industrial provider of the Nerve Monitoring device.

### 3.4. Typology approach

The typology approach in primary studies retrieved form meta-analyses/review is shown in Table 3. Only 2 out of 84 primary studies reported all the 3 items of the typology we developed; 9 of them reported 2 out of the 3 items, while 24 more stated only one of these typology items in their methods. Additionally, 49 out of 84 studies did not mention any information at all about the surgical technique. The analysis of the specific operational technical details, in cases which the procedure of thyroidectomy was described in detail, are reported thoroughly in Table 4. The categorization of the various descriptions of the RLN identification types and the reported dissection plane of thyroidectomy was found 34.52% (29 out of 84) and 13.09% (11 out of 84), respectively.

**Table 3:** Reported items, alone or in combination, of typology in primary studies (*n* = 84).

Items of typology	Number of studies (%)	References
Item 1: level of RLN identification technique	29 (34.52%)	(3, 4, 8, 13, 18, 20–22, 28–30, 34, 38–40, 43, 45–47, 52, 56, 68, 70–72, 75, 77, 78, 83)
Item 2: complete or partial exposure of the RLN	8 (9.52%)	(4, 8, 18, 22, 28, 39, 40, 45)
Item 3: reported technique of thyroidectomy (dissection plan)	11 (13.09%)	(3, 4, 6, 7, 22, 37, 46, 49, 58, 74, 77)
Combination of Items 1 and 2	6 (7.14%)	(8, 18, 28, 39, 40, 45)
Combination of Items 1 and 3	3 (3.57%)	(3, 46, 77)
Combination of Items 2 and 3	0 (0.00%)	
Combination of Items 1, 2 and 3	2 (2.38%)	(4, 22)
No reporting Items at all	49 (58.33%)	(1, 2, 5, 9–12, 14–17, 19, 23–27, 31–33, 35, 36, 41, 42, 44, 48, 50, 51, 53–55, 57, 59–67, 69, 73, 76, 79–82, 84)

The references are included in Supplementary Appendix 1.

**Table 4:** Categorization of the approach type: (1) to the RLN according to Goldenberg and Randolph (24), and (2) to the dissection technique of thyroidectomy according to Delbridge (17) and Gemsenjäger et al. (25).

1. Type (level) of approach of the RLN as concluded from the description in studies included in the meta-analyses/review.
Superior level (*n* = 5) - At the area of the ligament of Berry (3) - Five to ten millimeters before the entrance to larynx (8, 18) - Proximally near the cricothyroid joint after pericapsular dissection (13) - Lateral to the Berry ligament 5–10 mm before entry of the larynx (29) Lateral level (*n* = 15) - Tracheoesophageal groove (20, 21, 30, 47, 52, 68, 78) - Technique according to Harness (22) - Identified at the level of inferior thyroid artery or near the entry of the larynx (38, 46) - At the cross point with the inferior thyroid artery (56) - Two to 3 cm inferior to the lower border of the cricothyroid muscle to its laryngeal entrance (70, 71) - Superiorly to the inferior thyroid artery and exposed to larynx entry point (75) - In the middle third of its cervical course (77) Inferior level (*n* = 9) - Technique according to Lahey (4) - Low in the mediastinum (28) - Caudal to the lower lobe (34) - From the jugular triangle to the larynx entry (39, 40) - Dissected from below the inferior thyroid artery (43, 72) - Low in the neck (below the crossing with the inferior thyroid artery) (45) - In the inferior pole area traced to the cricothyroid membrane (83) Not reporting any information about the RLN approach level (*n* = 55) (1, 2, 5–7, 9–12, 14–17, 19, 23–27, 31–33, 35–37, 41, 42, 44, 48–51, 53–55, 57–67, 69, 73, 74, 76, 79–82, 84)
2. Technique of thyroidectomy (dissection plan) as stated in studies included in the meta-analyses/review.
Capsular (*n* = 2) (37, 49) Extracapsular (*n* = 5) - Extracapsular (4, 46, 77) - Total extracapsular (6, 7) Various descriptions (*n* = 4) - According to Harness (3, 22) - Capsular dissection after identifying the RLN (58) - Microdissection technique (74) Not reporting the preferred type of thyroid gland dissection (*n* = 73) (1, 2, 5, 8–21, 23–36, 38–45, 47, 48, 50–57, 59–73, 75, 76, 78–84)

The references are included in Supplementary Appendix 1.

Moreover, the screening of the primary studies' reference lists revealed that 9 studies (10.71%) [references 3, 4, 22, 37, 39, 40, 62, 71, 75 in Supplementary Appendix 1] included a reference supporting the initial identification type/level/approach of dissection of the RLN; 7 studies (8.33%) [references 3, 4, 22, 37, 48, 49, 75 in Supplementary Appendix 1] reported a citation that refers to the technique of thyroidectomy; none of them cited any article referring to the extent of exposure (total or partial) of RLN.

## 4. Discussion

Surgical technique of any operation, the essence of surgical art, should be described, in form of essential surgical steps, in any paper dealing with this issue (6, 40). In this study, we traced how the surgical technique of thyroidectomy is described in 84 original studies (Supplementary Appendix 1), in 12 meta-analyses (3, 31–41) and one systematic review (42) that compare and evaluate IONM versus VONA, regarding RLN injury. For this purpose, and considering that there has not been a similar publication to date, we fragmented the surgical technique at those points (items) which we believe that are crucial to thyroid surgery, in terms of RLN protection (24–27), developing a preliminary typology (6). We then constructed a framework for the reviews and meta-analyses in order to approach this typology in their included studies. Our results are in complete alignment with those deriving from studies of other surgical specialties (43, 44). Only in 2 of the 84 studies vague information about the identification approach of the RLN (item 1), the extent of nerve exposure (item 2) and the dissection plane of thyroidectomy (item 3) was provided (Table 3). Twenty-nine studies (34.52%) only briefly mentioned the level of initial identification approach of the nerve and none of them used the terminology for the types of RLN approaches, as described in classical textbooks (24), resulting in 18 completely different descriptions, all presented in Table 4. It also appears that the differentiation between extra-capsular and capsular technique, which affects the way of approaching and protecting the nerve (17, 45, 46), has not been established as an essential description of thyroidectomy in the literature. In our material only 11 out of 84 studies (13.09%) differentiated their dissection technique in terms of extracapsular or capsular approach during thyroidectomy (Table 4).

A standard and precisely described operational technique (47) reveals three important aspects of surgical knowledge. Firstly, it determines the methodological quality of any pertinent surgical study and promotes fidelity and generalizability (43, 48, 49). Secondly, it offers the opportunity to gain confidence in the particular operation revealing a network of valuable references (28) and substantial information structuralizing so the tacit knowledge, which characterizes surgery (11). Finally, it defines, in the course of the natural history of any procedure (1), those components of an operation, which are, will or have to be “gold standard”, clarifying basic principles for safe surgical practice (26).

Since 2008, there are guidelines for reporting surgical interventions in surgical studies of any design (5, 6, 50), and for the evaluation of new medical technology and devices as well (51). The appraisal of compliance to these reporting guidelines constitutes a meaningful synergy between meta-analyses/reviews and surgical studies (10). Therefore, we anticipated that in all meta-analyses/review the absence of typology or the heterogeneity produced from non-standardized techniques in surgical studies should be considered as a risk of intervention bias (19). Nonetheless, it was reported in only five (31, 33, 37, 39, 41). The phrase “the RLN is normally identified by palpation and dissection” in the meta-analysis by Cirocchi et al. (31) summarizes an overlooking and oversimplified approach to important aspects of a surgical technique which needs standardization (11). Furthermore, with the exception of two (33, 36), all the other meta-analyses/review incorporate in their comparative evaluation dissimilar surgical approaches (robotic, endoscopic-minimally invasive and conventional thyroid surgery) that involve totally different RLN handling and dissecting techniques during thyroidectomy. Additionally, no information or comments are noticed on the importance of standardization or reporting the type of RLN identification technique in recently published prospectively designed audits (52–55) and in articles about the history of thyroid surgery as well (56). Undoubtedly, the absence or inadequate reporting of surgical technique in surgical studies is not a recent finding (57). Only 5.95% [references 3, 4, 22, 37, 75 in Supplementary Appendix 1] of the studies refer to publications with diligent and precise descriptions of thyroidectomy. A superficial explanation could be that the omission of the description of the technique may be due to the limitation imposed by the publishers in terms of the number of words in a manuscript or because of the potential resulting plagiarism. However, the reporting trend is extremely low as shown in our study; forty-nine out of 84 studies (58.33%) did not mention any information at all about the surgical technique.

In the research field that confronts VONA with IONM, the importance of nerve dissection technique is essentially unique for both approaches (16, 17, 46, 47, 58). It bridges the two surgical strategies under comparison (59) and is the ultimate prerequisite for proper utilization of IONM (18). In fact, accurate monitoring is not feasible unless a nerve is visible (21) and according to Agha et al. “the decrease in RLN injury (with) IONM may be explained by the necessity of visual identification and surgical preparation of the recurrent laryngeal nerve.” (59). However, one will wonder, how exactly should the nerve be dissected, visualized and monitored, if selected so, and what are the basic principles that need to be followed in order to limit irreversible anatomical damage of RLN avoiding permanent VCP (60)? Unfortunately, the average reader cannot find any answer in the included studies and meta-analyses (61). In contrast, the technical principles of IONM are described thoroughly in all studies. These observations raise concerns about the peril of misconception that IONM could obviate the technical crucial act and craft of meticulous dissection, preparation, identification and visualization of the nerve (62). The consequences of this misleading conception become particularly noteworthy if we realize that 50%–95% of surgeons perform annually one to fifteen thyroidectomies, carrying out 70% of all thyroidectomies per year in the US (63). Hence, thyroidectomy, as many other procedures in surgical studies (5, 13, 64, 65), is not standardized (66). All those small and numerous steps-items that ensure the atraumatic dissection of the RLN must be defined, agreed and ultimately synthesized to a reproducible and teachable operation (66). This heuristics perspective in thyroidectomy (66) can build the platform of typology in thyroid surgery, for which we provided a prototype attempt.

There are several limitations in our study that should be mentioned. We have arbitrarily tried to create a typology which would ensure an adequate description of the technique to be followed in each thyroidectomy, allowing comparisons. However, our aim was to introduce in the international literature a discussion aimed at clearly defining a typology for thyroidectomy, rather than describing it in detail. This could be only achieved after consensus in a panel of expert endocrine surgeons in order to be widely accepted. We focused solely in technical details relevant to RLN isolating our typology from technical issues related to safeguarding of the external branch of the superior laryngeal nerve and the parathyroid glands. These perspectives should be also evaluated. We also did not refer to the potential impact of IONM on surgeon's performance and his or her level of experience during thyroidectomy (67), reported disadvantages (68) and important advancements of this technology (18). Another weak point is that we have completely detached the identification technique and handling of the RLN from the extent of thyroidectomy (69) and from conditions that render the nerve vulnerable to injury, such as the presence of RLN extra-laryngeal branching (70), the use of energy devices (71) and inappropriate traction on the thyroid lobe (72). All these aspects should be also considered for a typology in terms of RLN management during thyroid surgery.

On the other hand, we have documented for the first time that basic components of the surgical technique in thyroidectomy are not described in studies comparing VONA to IONM. This absence of typology is responsible, among other reasons, for the conflicting and ambiguous results of meta-analyses, leaving the reader with more questions than answers and the recommendation that “more RCTs are needed” (9). Unresolved remain also two issues regarding the correlation between industry sponsorship and research findings as pointed out also by other authors (30): the exact definition of what constitute the conflict of interests and the standardization of reporting these conflicts. This is of outmost importance if an industrial produced device has to be evaluated.

In conclusion, a transparent description of the surgical technique is the typology that is missing from surgical research nowadays and should find its place in studies that compare and evaluate VONA versus IONM, in terms of avoiding permanent RLN injury. Research efforts must shift from this debate to a typology project of “standardizing and reporting” emphasizing technical aspects of safety. A bright example of this perspective is given for another very common procedure in general surgery that is cholecystectomy by the American Task Force for safe cholecystectomy (73). This typology, in synergy with a coherent framework approach by the meta-analyses and reviews focusing on items of surgical technique, enables surgeons to translate the vast amount of data generated from clinical research into knowledge that can form principles for a safe surgical practice.

## References

[1] McCulloch P. Developing appropriate methodology for the study of surgical techniques. J R Soc Med. (2009) 102(2):51–5. 10.1258/jrsm.2008.080308 19208868 PMC2642873

[2] Robinson NB Fremes S Hameed I Rahouma M Weidenmann V Demetres M Characteristics of randomized clinical trials in surgery from 2008 to 2020: a systematic review. JAMA Netw Open. (2021) 4(6):e2114494. 10.1001/jamanetworkopen.2021.14494 34190996 PMC8246313

[3] Davey MG Cleere EF Lowery AJ Kerin MJ. Intraoperative recurrent laryngeal nerve monitoring versus visualisation alone—a systematic review and meta-analysis of randomized controlled trials. Am J Surg. (2022) 224(3):836–41. 10.1016/j.amjsurg.2022.03.036 35422329

[4] Sanabria A Kowalski LP Nixon I Angelos P Shaha A Owen RP Methodological quality of systematic reviews of intraoperative neuromonitoring in thyroidectomy: a systematic review. JAMA Otolaryngol Head Neck Surg. (2019) 145(6):563–73. 10.1001/jamaoto.2019.0092 30973598

[5] Zhang K Ma Y Shi Q Wu J Shen J He Y Developing the surgical technique reporting checklist and standards: a study protocol. Gland Surg. (2021) 10(8):2591–9. 10.21037/gs-21-312 34527570 PMC8411094

[6] Blencowe NS Mills N Cook JA Donovan JL Rogers CA Whiting P Standardizing and monitoring the delivery of surgical interventions in randomized clinical trials. Br J Surg. (2016) 103(10):1377–84. 10.1002/bjs.10254 27462835 PMC5132147

[7] Jacquier I Boutron I Moher D Roy C Ravaud P. The reporting of randomized clinical trials using a surgical intervention is in need of immediate improvement: a systematic review. Ann Surg. (2006) 244(5):677–83. 10.1097/01.sla.0000242707.44007.80 17060758 PMC1856606

[8] Lawrentschuk N McCall J Güller U. Critical appraisal of meta-analyses: an introductory guide for the practicing surgeon. Patient Saf Surg. (2009) 3(1):16. 10.1186/1754-9493-3-16 19624816 PMC2731030

[9] Agha R Cooper D Muir G. The reporting quality of randomised controlled trials in surgery: a systematic review. Int J Surg. (2007) 5(6):413–22. 10.1016/j.ijsu.2007.06.002 18029237

[10] Diener MK Simon T Büchler MW Seiler CM. Surgical evaluation and knowledge transfer–methods of clinical research in surgery. Langenbecks Arch Surg. (2012) 397(8):1193–9. 10.1007/s00423-011-0775-x 21424797

[11] Lilford R Braunholtz D Harris J Gill T. Trials in surgery. Br J Surg. (2004) 91(1):6–16. 10.1002/bjs.4418 14716788

[12] McCulloch P Taylor I Sasako M Lovett B Griffin D. Randomised trials in surgery: problems and possible solutions. Br Med J. (2002) 324(7351):1448–51. 10.1136/bmj.324.7351.1448 12065273 PMC1123389

[13] Bailey CW Crosby LD Johnson B Lokey JS. Assessment of time and cost of anesthesia with versus without recurrent laryngeal nerve monitoring in patients undergoing total thyroidectomy. Am Surg. (2011) 77(8):E158–9. 10.1177/000313481107700803 21944499

[14] Meakins JL. Innovation in surgery: the rules of evidence. Am J Surg. (2002) 183(4):399–405. 10.1016/s0002-9610(02)00825-5 11975927

[15] Bothra S Sabaretnam M Kannujia A Chand G Agarwal G Mishra SK Patient, thyroid, and surgeon related factors that make thyroidectomy difficult-cohort study. Ann Med Surg. (2020) 49:14–8. 10.1016/j.amsu.2019.11.010 PMC6909039 31871677

[16] Lenay-Pinon D Biet-Hornstein A Strunski V Page C. The circumstances in which recurrent laryngeal nerve palsy occurs after surgery for benign thyroid disease: a retrospective study of 1026 patients. J Laryngol Otol. (2021) 135(7):640–3. 10.1017/s0022215121001493 34120661

[17] Delbridge L. Total thyroidectomy: the evolution of surgical technique. ANZ J Surg. (2003) 73(9):761–8. 10.1046/j.1445-2197.2003.02756.x 12956795

[18] Schneider R Machens A Lorenz K Dralle H. Intraoperative nerve monitoring in thyroid surgery-shifting current paradigms. Gland Surg. (2020) 9(Suppl 2):S120–s8. 10.21037/gs.2019.11.04 32175252 PMC7044089

[19] Kalkum E Klotz R Seide S Hüttner FJ Kowalewski KF Nickel F Systematic reviews in surgery-recommendations from the study center of the German society of surgery. Langenbecks Arch Surg. (2021) 406(6):1723–31. 10.1007/s00423-021-02204-x 34129108 PMC8481197

[20] Henry BM Graves MJ Vikse J Sanna B Pękala PA Walocha JA The current state of intermittent intraoperative neural monitoring for prevention of recurrent laryngeal nerve injury during thyroidectomy: a prisma-compliant systematic review of overlapping meta-analyses. Langenbecks Arch Surg. (2017) 402(4):663–73. 10.1007/s00423-017-1580-y 28378238 PMC5437188

[21] Kim DH Kim SW Hwang SH. Intraoperative neural monitoring for early vocal cord function assessment after thyroid surgery: a systematic review and meta-analysis. World J Surg. (2021) 45(11):3320–7. 10.1007/s00268-021-06225-x 34191086

[22] Ku D Hui M Cheung P Chow O Smith M Riffat F Meta-analysis on continuous nerve monitoring in thyroidectomies. Head Neck. (2021) 43(12):3966–78. 10.1002/hed.26828 34342380

[23] Page MJ McKenzie JE Bossuyt PM Boutron I Hoffmann TC Mulrow CD The prisma 2020 statement: an updated guideline for reporting systematic reviews. Br Med J. (2021) 372:n71. 10.1136/bmj.n71 33782057 PMC8005924

[24] David Goldenberg GWR. The recurrent laryngeal nerve. In: Miccoli DJT P Minuto MN Seybt MW, editors. Thyroid surgery preventing and managing complications. West Sussex: Wiley-Blackwell (2013). p. 119–27.

[25] Gemsenjaeger E. Capsular dissection. In: Principles, practice, and clinical cases Stuttgart. New York: Thieme (2009). p. 9–23.

[26] Hermann M Alk G Roka R Glaser K Freissmuth M. Laryngeal recurrent nerve injury in surgery for benign thyroid diseases: effect of nerve dissection and impact of individual surgeon in more than 27,000 nerves at risk. Ann Surg. (2002) 235(2):261–8. 10.1097/00000658-200202000-00015 11807367 PMC1422423

[27] Veyseller B Aksoy F Yildirim YS Karatas A Ozturan O. Effect of recurrent laryngeal nerve identification technique in thyroidectomy on recurrent laryngeal nerve paralysis and hypoparathyroidism. Arch Otolaryngol Head Neck Surg. (2011) 137(9):897–900. 10.1001/archoto.2011.134 21844405

[28] Ngatuvai M Autrey C McKenny M Elkbuli A. Significance and implications of accurate and proper citations in clinical research studies. Ann Med Surg. (2021) 72:102841. 10.1016/j.amsu.2021.102841 PMC8712974 34992774

[29] Probst P Knebel P Grummich K Tenckhoff S Ulrich A Büchler MW Industry bias in randomized controlled trials in general and abdominal surgery: an empirical study. Ann Surg. (2016) 264(1):87–92. 10.1097/sla.0000000000001372 26465782

[30] Probst P Grummich K Klaiber U Knebel P Ulrich A Büchler MW Conflicts of interest in randomised controlled surgical trials: systematic review and qualitative and quantitative analysis. Innov Surg Sci. (2016) 1(1):33–9. 10.1515/iss-2016-0001 31579716 PMC6753986

[31] Cirocchi R Arezzo A D'Andrea V Abraha I Popivanov GI Avenia N Intraoperative neuromonitoring versus visual nerve identification for prevention of recurrent laryngeal nerve injury in adults undergoing thyroid surgery. Cochrane Database Syst Rev. (2019) 1(1):Cd012483. 10.1002/14651858.CD012483.pub2 30659577 PMC6353246

[32] Bai B Chen W. Protective effects of intraoperative nerve monitoring (ionm) for recurrent laryngeal nerve injury in thyroidectomy: meta-analysis. Sci Rep. (2018) 8(1):7761. 10.1038/s41598-018-26219-5 29773852 PMC5958090

[33] Wong KP Mak KL Wong CKH Lang BHH. Systematic review and meta-analysis on intra-operative neuro-monitoring in high-risk thyroidectomy. Int J Surg. (2017) 38:21–30. 10.1016/j.ijsu.2016.12.039 28034775

[34] Yang S Zhou L Lu Z Ma B Ji Q Wang Y. Systematic review with meta-analysis of intraoperative neuromonitoring during thyroidectomy. Int J Surg. (2017) 39:104–13. 10.1016/j.ijsu.2017.01.086 28130189

[35] Lombardi CP Carnassale G Damiani G Acampora A Raffaelli M De Crea C The final countdown: is intraoperative, intermittent neuromonitoring really useful in preventing permanent nerve palsy? Evidence from a meta-analysis. Surgery. (2016) 160(6):1693–706. 10.1016/j.surg.2016.06.049 27566947

[36] Pardal-Refoyo JL Ochoa-Sangrador C. Bilateral recurrent laryngeal nerve injury in total thyroidectomy with or without intraoperative neuromonitoring. Systematic review and meta-analysis. Acta Otorrinolaringol Esp. (2016) 67(2):66–74. 10.1016/j.otorri.2015.02.001 26025358

[37] Pisanu A Porceddu G Podda M Cois A Uccheddu A. Systematic review with meta-analysis of studies comparing intraoperative neuromonitoring of recurrent laryngeal nerves versus visualization alone during thyroidectomy. J Surg Res. (2014) 188(1):152–61. 10.1016/j.jss.2013.12.022 24433869

[38] Rulli F Ambrogi V Dionigi G Amirhassankhani S Mineo TC Ottaviani F Meta-analysis of recurrent laryngeal nerve injury in thyroid surgery with or without intraoperative nerve monitoring. Acta Otorhinolaryngol Ital. (2014) 34(4):223–9. 25210215 PMC4157532

[39] Higgins TS Gupta R Ketcham AS Sataloff RT Wadsworth JT Sinacori JT. Recurrent laryngeal nerve monitoring versus identification alone on post-thyroidectomy true vocal fold palsy: a meta-analysis. Laryngoscope. (2011) 121(5):1009–17. 10.1002/lary.21578 21520117

[40] Sanabria A Ramirez A Kowalski LP Silver CE Shaha AR Owen RP Neuromonitoring in thyroidectomy: a meta-analysis of effectiveness from randomized controlled trials. Eur Arch Otorhinolaryngol. (2013) 270(8):2175–89. 10.1007/s00405-013-2557-2 23681545

[41] Zheng S Xu Z Wei Y Zeng M He J. Effect of intraoperative neuromonitoring on recurrent laryngeal nerve palsy rates after thyroid surgery–a meta-analysis. J Formos Med Assoc. (2013) 112(8):463–72. 10.1016/j.jfma.2012.03.003 24016611

[42] Malik R Linos D. Intraoperative neuromonitoring in thyroid surgery: a systematic review. World J Surg. (2016) 40(8):2051–8. 10.1007/s00268-016-3594-y 27329143

[43] Torgerson T Johnson AL Jellison S Tanghetti M Langley JM Nguyen LHP Reporting of clinical trial interventions published in leading otolaryngology-head and neck surgery journals. Laryngoscope. (2020) 130(9):E507–e14. 10.1002/lary.28404 31747063

[44] Anderson JM Stafford A Jellison S Vassar M. Intervention reporting of published trials is insufficient in orthopaedic surgery journals: application of the template for intervention description and replication checklist. Arthrosc Sports Med Rehabil. (2021) 3(3):e619–e27. 10.1016/j.asmr.2020.09.019 34195624 PMC8220564

[45] Gemsenjäger E. Das Bild der grenzlamelle in der schilddrüsenchirurgie. Der Chirurg. (2009) 80(12):1165. 10.1007/s00104-009-1849-y 19936991

[46] Hayward NJ Grodski S Yeung M Johnson WR Serpell J. Recurrent laryngeal nerve injury in thyroid surgery: a review. ANZ J Surg. (2013) 83(1-2):15–21. 10.1111/j.1445-2197.2012.06247.x 22989215

[47] Shindo M Chheda NN. Incidence of vocal cord paralysis with and without recurrent laryngeal nerve monitoring during thyroidectomy. Arch Otolaryngol Head Neck Surg. (2007) 133(5):481–5. 10.1001/archotol.133.5.481 17520762

[48] Wenner DM Brody BA Jarman AF Kolman JM Wray NP Ashton CM. Do surgical trials meet the scientific standards for clinical trials? J Am Coll Surg. (2012) 215(5):722–30. 10.1016/j.jamcollsurg.2012.06.018 22819638 PMC3478478

[49] Stirrat GM. Ethics and evidence based surgery. J Med Ethics. (2004) 30(2):160–5. 10.1136/jme.2003.007054 15082810 PMC1733841

[50] Boutron I Altman DG Moher D Schulz KF Ravaud P. Consort statement for randomized trials of nonpharmacologic treatments: a 2017 update and a consort extension for nonpharmacologic trial abstracts. Ann Intern Med. (2017) 167(1):40–7. 10.7326/m17-0046 28630973

[51] McCulloch P Altman DG Campbell WB Flum DR Glasziou P Marshall JC No surgical innovation without evaluation: the ideal recommendations. Lancet. (2009) 374(9695):1105–12. 10.1016/s0140-6736(09)61116-8 19782876

[52] Gunn A Oyekunle T Stang M Kazaure H Scheri R. Recurrent laryngeal nerve injury after thyroid surgery: an analysis of 11,370 patients. J Surg Res. (2020) 255:42–9. 10.1016/j.jss.2020.05.017 32540579

[53] Abdelhamid A Aspinall S. Intraoperative nerve monitoring in thyroid surgery: analysis of United Kingdom registry of endocrine and thyroid surgery database. Br J Surg. (2021) 108(2):182–7. 10.1093/bjs/znaa081 33711146

[54] Liu JB Sosa JA Grogan RH Liu Y Cohen ME Ko CY Variation of thyroidectomy-specific outcomes among hospitals and their association with risk adjustment and hospital performance. JAMA Surg. (2018) 153(1):e174593. 10.1001/jamasurg.2017.4593 29188293 PMC5833609

[55] Mahoney RC Vossler JD Murayama KM Woodruff SL. Predictors and consequences of recurrent laryngeal nerve injury during open thyroidectomy: an American college of surgeons national surgical quality improvement project database analysis. Am J Surg. (2021) 221(1):122–6. 10.1016/j.amjsurg.2020.07.023 32811620

[56] Christoforides C Dionigi G Vasileiou I Vamvakidis K. A historical account for thyroid surgery. J Endocr Surg. (2018) 18(1):1–9. 10.16956/jes.2018.18.1.1

[57] Jatzko GR Lisborg PH Müller MG Wette VM. Recurrent nerve palsy after thyroid operations–principal nerve identification and a literature review. Surgery. (1994) 115(2):139–44. 8310401

[58] Snyder SK Sigmond BR Lairmore TC Govednik-Horny CM Janicek AK Jupiter DC. The long-term impact of routine intraoperative nerve monitoring during thyroid and parathyroid surgery. Surgery. (2013) 154(4):704–11. 10.1016/j.surg.2013.06.039 24008089

[59] Agha A Glockzin G Ghali N Iesalnieks I Schlitt HJ. Surgical treatment of substernal goiter: an analysis of 59 patients. Surg Today. (2008) 38(6):505–11. 10.1007/s00595-007-3659-5 18516529

[60] Hisham AN Lukman MR. Recurrent laryngeal nerve in thyroid surgery: a critical appraisal. ANZ J Surg. (2002) 72(12):887–9. 10.1046/j.1445-2197.2002.02578.x 12485227

[61] Meshikhes AW. Evidence-based surgery: the obstacles and solutions. Int J Surg. (2015) 18:159–62. 10.1016/j.ijsu.2015.04.071 25934416

[62] Delbridge L Gough I Lisewski D Middleton P Miller J Parkyn R Consensus statements in surgery: intra-operative neural monitoring for thyroid surgery. ANZ J Surg. (2015) 85:5–7. 10.1111/ans.12966 25759889

[63] Adam MA Thomas S Youngwirth L Hyslop T Reed SD Scheri RP Is there a minimum number of thyroidectomies a surgeon should perform to optimize patient outcomes? Ann Surg. (2017) 265(2):402–7. 10.1097/sla.0000000000001688 28059969

[64] Lawrence K McWhinnie D Collin J Morris P. Surgical evaluation. Br J Surg. (1994) 81(9):1390–2. 10.1002/bjs.1800810950 7953428

[65] Pegg DJ. Evaluating new surgical procedures. Hip replacements come in at least 10(11) varieties. Br Med J. (1996) 312(7031):637. 10.1136/bmj.312.7031.637 PMC2350425 8595353

[66] Serpell JW Grodski S Yeung M Swann J Kemp S Johnson W. Hemithyroidectomy: a heuristics perspective. ANZ J Surg. (2008) 78(12):1122–7. 10.1111/j.1445-2197.2008.04764.x 19087056

[67] Duclos A Lifante JC Ducarroz S Soardo P Colin C Peix JL. Influence of intraoperative neuromonitoring on surgeons’ technique during thyroidectomy. World J Surg. (2011) 35(4):773–8. 10.1007/s00268-011-0963-4 21267565

[68] Terris DJ Chaung K Duke WS. Continuous vagal nerve monitoring is dangerous and should not routinely be done during thyroid surgery. World J Surg. (2015) 39(10):2471–6. 10.1007/s00268-015-3139-9 26138874

[69] Landerholm K Wasner AM Järhult J. Incidence and risk factors for injuries to the recurrent laryngeal nerve during neck surgery in the moderate-volume setting. Langenbecks Arch Surg. (2014) 399(4):509–15. 10.1007/s00423-013-1154-6 24402457

[70] Sancho JJ Pascual-Damieta M Pereira JA Carrera MJ Fontané J Sitges-Serra A. Risk factors for transient vocal cord palsy after thyroidectomy. Br J Surg. (2008) 95(8):961–7. 10.1002/bjs.6173 18618893

[71] Chen J Tang Z. A commentary on “mechanisms of recurrent laryngeal nerve injury near the nerve entry point during thyroid surgery: a retrospective cohort study” (Int J Surg 2020; 83:125-130). Int J Surg. (2021) 92:106040. 10.1016/j.ijsu.2021.106040 34339884

[72] Brauckhoff K Vik R Sandvik L Heimdal JH Aas T Biermann M Impact of EMG changes in continuous vagal nerve monitoring in high-risk endocrine neck surgery. World J Surg. (2016) 40(3):672–80. 10.1007/s00268-015-3368-y 26678490 PMC4746223

[73] Brunt LM Deziel DJ Telem DA Strasberg SM Aggarwal R Asbun H Safe cholecystectomy multi-society practice guideline and state of the art consensus conference on prevention of bile duct injury during cholecystectomy. Ann Surg. (2020) 272(1):3–23. 10.1097/sla.0000000000003791 32404658

