# Supplementary material for: Visualization of the recurrent laryngeal nerve alone versus intraoperative nerve monitoring in primary thyroidectomy: a framework approach to a missing typology
Source: Front Surg. 2023 Jul 24;10:1176511. doi: 10.3389/fsurg.2023.1176511 (PMC10406577; doi:10.3389/fsurg.2023.1176511)
Supplement: Supplementary file 1 [file Table1.docx]

Supplementary Material

Visualization of the Recurrent Laryngeal Nerve Alone Versus Intraoperative Nerve Monitoring in Primary Thyroidectomy. A Framework Approach to a Missing Typology.

**Dimitris Papagoras, Georgios Tzikos*, Gerasimos Douridas, Polyvios Arseniou, Dimitrios Panagiotou, Maria Kanara, Theodosios Papavramidis**

*** Correspondence:** Georgios Tzikos: tzikos_giorgos@outlook.com

# Supplementary Data

**Appendix 1.** References of included studies, extracted from meta-analyses/review (31-42).

1. Agha A, Glockzin G, Ghali N, Iesalnieks I, Schlitt HJ. Surgical Treatment of Substernal Goiter: An Analysis of 59 Patients. *Surg Today* (2008) 38(6):505-11. Epub 2008/06/03. doi: 10.1007/s00595-007-3659-5.

2. Alesina PF, Hinrichs J, Meier B, Cho EY, Bolli M, Walz MK. Intraoperative Neuromonitoring for Surgical Training in Thyroid Surgery: Its Routine Use Allows a Safe Operation Instead of Lack of Experienced Mentoring. *World J Surg* (2014) 38(3):592-8. Epub 2013/12/07. doi: 10.1007/s00268-013-2372-3.

3. Anuwong A, Lavazza M, Kim HY, Wu CW, Rausei S, Pappalardo V, et al. Recurrent Laryngeal Nerve Management in Thyroid Surgery: Consequences of Routine Visualization, Application of Intermittent, Standardized and Continuous Nerve Monitoring. *Updates Surg* (2016) 68(4):331-41. Epub 2016/09/22. doi: 10.1007/s13304-016-0393-9.

4. Ardito G, Revelli L, D'Alatri L, Lerro V, Guidi ML, Ardito F. Revisited Anatomy of the Recurrent Laryngeal Nerves. *Am J Surg* (2004) 187(2):249-53. Epub 2004/02/11. doi: 10.1016/j.amjsurg.2003.11.001.

5. Barczyński M, Konturek A, Cichoń S. Randomized Clinical Trial of Visualization Versus Neuromonitoring of Recurrent Laryngeal Nerves During Thyroidectomy. *Br J Surg* (2009) 96(3):240-6. Epub 2009/01/30. doi: 10.1002/bjs.6417.

6. Barczyński M, Konturek A, Stopa M, Honowska A, Nowak W. Randomized Controlled Trial of Visualization Versus Neuromonitoring of the External Branch of the Superior Laryngeal Nerve During Thyroidectomy. *World J Surg* (2012) 36(6):1340-7. Epub 2012/03/10. doi: 10.1007/s00268-012-1547-7.

7. Barczyński M, Konturek A, Stopa M, Hubalewska-Dydejczyk A, Richter P, Nowak W. Clinical Value of Intraoperative Neuromonitoring of the Recurrent Laryngeal Nerves in Improving Outcomes of Surgery for Well-Differentiated Thyroid Cancer. *Pol Przegl Chir* (2011) 83(4):196-203. Epub 2011/12/15. doi: 10.2478/v10035-011-0030-8.

8. Beldi G, Kinsbergen T, Schlumpf R. Evaluation of Intraoperative Recurrent Nerve Monitoring in Thyroid Surgery. *World J Surg* (2004) 28(6):589-91. Epub 2004/09/16. doi: 10.1007/s00268-004-7226-6.

9. Bergenfelz A, Jansson S, Kristoffersson A, Mårtensson H, Reihnér E, Wallin G, et al. Complications to Thyroid Surgery: Results as Reported in a Database from a Multicenter Audit Comprising 3,660 Patients. *Langenbecks Arch Surg* (2008) 393(5):667-73. Epub 2008/07/18. doi: 10.1007/s00423-008-0366-7.

10. Bhattacharyya N, Fried MP. Assessment of the Morbidity and Complications of Total Thyroidectomy. *Arch Otolaryngol Head Neck Surg* (2002) 128(4):389-92. Epub 2002/04/03. doi: 10.1001/archotol.128.4.389.

11. Brajcich BC, McHenry CR. The Utility of Intraoperative Nerve Monitoring During Thyroid Surgery. *J Surg Res* (2016) 204(1):29-33. Epub 2016/07/28. doi: 10.1016/j.jss.2016.04.039.

12. Brauckhoff M, Gimm O, Thanh PN, Brauckhoff K, Ukkat J, Thomusch O, et al. First Experiences in Intraoperative Neurostimulation of the Recurrent Laryngeal Nerve During Thyroid Surgery of Children and Adolescents. *J Pediatr Surg* (2002) 37(10):1414-8. Epub 2002/10/16. doi: 10.1053/jpsu.2002.35403.

13. Brennan J, Moore EJ, Shuler KJ. Prospective Analysis of the Efficacy of Continuous Intraoperative Nerve Monitoring During Thyroidectomy, Parathyroidectomy, and Parotidectomy. *Otolaryngol Head Neck Surg* (2001) 124(5):537-43. Epub 2001/05/05. doi: 10.1067/mhn.2001.115402.

14. Calò PG, Medas F, Erdas E, Pittau MR, Demontis R, Pisano G, et al. Role of Intraoperative Neuromonitoring of Recurrent Laryngeal Nerves in the Outcomes of Surgery for Thyroid Cancer. *International Journal of Surgery* (2014) 12:S213-S7. doi: <https://doi.org/10.1016/j.ijsu.2014.05.003>.

15. Calò PG, Medas F, Gordini L, Podda F, Erdas E, Pisano G, et al. Interpretation of Intraoperative Recurrent Laryngeal Nerve Monitoring Signals: The Importance of a Correct Standardization. *International Journal of Surgery* (2016) 28:S54-S8. doi: <https://doi.org/10.1016/j.ijsu.2015.12.039>.

16. Calò PG, Pisano G, Medas F, Pittau MR, Gordini L, Demontis R, et al. Identification Alone Versus Intraoperative Neuromonitoring of the Recurrent Laryngeal Nerve During Thyroid Surgery: Experience of 2034 Consecutive Patients. *J Otolaryngol Head Neck Surg* (2014) 43(1):16-. doi: 10.1186/1916-0216-43-16.

17. Calò PG, Pisano G, Medas F, Tatti A, Pittau MR, Demontis R, et al. Intraoperative Recurrent Laryngeal Nerve Monitoring in Thyroid Surgery: Is It Really Useful? *Clin Ter* (2013) 164(3):e193-8. Epub 2013/07/23. doi: 10.7417/ct.2013.1567.

18. Cavicchi O, Caliceti U, Fernandez IJ, Macrì G, Di Lieto C, Marcantoni A, et al. The Value of Neurostimulation and Intraoperative Nerve Monitoring of Inferior Laryngeal Nerve in Thyroid Surgery. *Otolaryngol Head Neck Surg* (2009) 140(6):866-70. Epub 2009/05/27. doi: 10.1016/j.otohns.2008.12.047.

19. Cernea CR, Brandão LG, Hojaij FC, De Carlucci D, Jr., Brandão J, Cavalheiro B, et al. Negative and Positive Predictive Values of Nerve Monitoring in Thyroidectomy. *Head Neck* (2012) 34(2):175-9. Epub 2011/03/18. doi: 10.1002/hed.21695.

20. Chan WF, Lang BH, Lo CY. The Role of Intraoperative Neuromonitoring of Recurrent Laryngeal Nerve During Thyroidectomy: A Comparative Study on 1000 Nerves at Risk. *Surgery* (2006) 140(6):866-72; discussion 72-3. Epub 2006/12/26. doi: 10.1016/j.surg.2006.07.017.

21. Chan WF, Lo CY. Pitfalls of Intraoperative Neuromonitoring for Predicting Postoperative Recurrent Laryngeal Nerve Function During Thyroidectomy. *World J Surg* (2006) 30(5):806-12. Epub 2006/05/09. doi: 10.1007/s00268-005-0355-8.

22. Chiang FY, Lu IC, Kuo WR, Lee KW, Chang NC, Wu CW. The Mechanism of Recurrent Laryngeal Nerve Injury During Thyroid Surgery--the Application of Intraoperative Neuromonitoring. *Surgery* (2008) 143(6):743-9. Epub 2008/06/14. doi: 10.1016/j.surg.2008.02.006.

23. Chiang FY, Wang LF, Huang YF, Lee KW, Kuo WR. Recurrent Laryngeal Nerve Palsy after Thyroidectomy with Routine Identification of the Recurrent Laryngeal Nerve. *Surgery* (2005) 137(3):342-7. Epub 2005/03/05. doi: 10.1016/j.surg.2004.09.008.

24. de Danschutter SJR, Schreinemakers JMJ, Smit LHM, van der Laan L, Nuytinck HKS. Thyroid Surgery and the Usefulness of Intraoperative Neuromonitoring, a Single Center Study. *Journal of Investigative Surgery* (2015) 28(2):86-94. doi: 10.3109/08941939.2014.975876.

25. De Falco M, Santangelo G, Del Giudice S, Gallucci F, Parmeggiani U. Double Probe Intraoperative Neuromonitoring with a Standardized Method in Thyroid Surgery. *Int J Surg* (2014) 12 Suppl 1:S140-4. Epub 2014/05/28. doi: 10.1016/j.ijsu.2014.05.026.

26. Dionigi G, Boni L, Rovera F, Rausei S, Castelnuovo P, Dionigi R. Postoperative Laryngoscopy in Thyroid Surgery: Proper Timing to Detect Recurrent Laryngeal Nerve Injury. *Langenbecks Arch Surg* (2010) 395(4):327-31. Epub 2009/12/17. doi: 10.1007/s00423-009-0581-x.

27. Dralle H, Sekulla C, Haerting J, Timmermann W, Neumann HJ, Kruse E, et al. Risk Factors of Paralysis and Functional Outcome after Recurrent Laryngeal Nerve Monitoring in Thyroid Surgery. *Surgery* (2004) 136(6):1310-22. Epub 2005/01/20. doi: 10.1016/j.surg.2004.07.018.

28. Duclos A, Lifante JC, Ducarroz S, Soardo P, Colin C, Peix JL. Influence of Intraoperative Neuromonitoring on Surgeons' Technique During Thyroidectomy. *World J Surg* (2011) 35(4):773-8. Epub 2011/01/27. doi: 10.1007/s00268-011-0963-4.

29. Eltzschig HK, Posner M, Moore FD, Jr. The Use of Readily Available Equipment in a Simple Method for Intraoperative Monitoring of Recurrent Laryngeal Nerve Function During Thyroid Surgery: Initial Experience with More Than 300 Cases. *Arch Surg* (2002) 137(4):452-6; discussion 6-7. Epub 2002/04/03. doi: 10.1001/archsurg.137.4.452.

30. Erçetin C, Şahbaz A, Acar S, Tutal F, Aksakal N, Sarı S, et al. Is Intraoperative Nerve Monitoring Useful for Surgical Training in Thyroid Surgery? *Turk J Surg* (2019) 35(4):259-64. Epub 2018/11/20. doi: 10.5578/turkjsurg.4281.

31. Frattini F, Mangano A, Boni L, Rausei S, Biondi A, Dionigi G. Intraoperative Neuromonitoring for Thyroid Malignancy Surgery: Technical Notes and Results from a Retrospective Series. *Updates Surg* (2010) 62(3-4):183-7. Epub 2010/12/15. doi: 10.1007/s13304-010-0036-5.

32. Gardner GM, Smith MM, Yaremchuk KL, Peterson EL. The Cost of Vocal Fold Paralysis after Thyroidectomy. *Laryngoscope* (2013) 123(6):1455-63. Epub 2013/05/25. doi: 10.1002/lary.23548.

33. Genther DJ, Kandil EH, Noureldine SI, Tufano RP. Correlation of Final Evoked Potential Amplitudes on Intraoperative Electromyography of the Recurrent Laryngeal Nerve with Immediate Postoperative Vocal Fold Function after Thyroid and Parathyroid Surgery. *JAMA Otolaryngol Head Neck Surg* (2014) 140(2):124-8. Epub 2014/01/05. doi: 10.1001/jamaoto.2013.6139.

34. Goretzki PE, Schwarz K, Brinkmann J, Wirowski D, Lammers BJ. The Impact of Intraoperative Neuromonitoring (Ionm) on Surgical Strategy in Bilateral Thyroid Diseases: Is It Worth the Effort? *World J Surg* (2010) 34(6):1274-84. Epub 2010/02/10. doi: 10.1007/s00268-009-0353-3.

35. Gremillion G, Fatakia A, Dornelles A, Amedee RG. Intraoperative Recurrent Laryngeal Nerve Monitoring in Thyroid Surgery: Is It Worth the Cost? *Ochsner J* (2012) 12(4):363-6.

36. Hamelmann W, Meyer T, Timm S, Timmermann W. Kritische Beurteilung Und Fehlermöglichkeiten Des Intraoperativen Neuromonitoring (Ionm) Bei Operationen an Der Schilddrüse. *Zentralblatt Fur Chirurgie* (2002) 127:409-13.

37. Hayward NJ, Grodski S, Yeung M, Johnson WR, Serpell J. Recurrent Laryngeal Nerve Injury in Thyroid Surgery: A Review. *ANZ J Surg* (2013) 83(1-2):15-21. Epub 2012/09/20. doi: 10.1111/j.1445-2197.2012.06247.x.

38. Hemmerling TM, Schmidt J, Bosert C, Jacobi KE, Klein P. Intraoperative Monitoring of the Recurrent Laryngeal Nerve in 151 Consecutive Patients Undergoing Thyroid Surgery. *Anesth Analg* (2001) 93(2):396-9 , 3rd contents page. Epub 2001/07/28. doi: 10.1097/00000539-200108000-00032.

39. Hermann M, Alk G, Roka R, Glaser K, Freissmuth M. Laryngeal Recurrent Nerve Injury in Surgery for Benign Thyroid Diseases: Effect of Nerve Dissection and Impact of Individual Surgeon in More Than 27,000 Nerves at Risk. *Ann Surg* (2002) 235(2):261-8. doi: 10.1097/00000658-200202000-00015.

40. Hermann M, Hellebart C, Freissmuth M. Neuromonitoring in Thyroid Surgery: Prospective Evaluation of Intraoperative Electrophysiological Responses for the Prediction of Recurrent Laryngeal Nerve Injury. *Ann Surg* (2004) 240(1):9-17. Epub 2004/06/24. doi: 10.1097/01.sla.0000132260.34503.02.

41. Herranz-González J, Gavilán J, Matínez-Vidal J, Gavilán C. Complications Following Thyroid Surgery. *Arch Otolaryngol Head Neck Surg* (1991) 117(5):516-8. Epub 1991/05/01. doi: 10.1001/archotol.1991.01870170062014.

42. Horn D, Rötzscher VM. Intraoperative Electromyogram Monitoring of the Recurrent Laryngeal Nerve: Experience with an Intralaryngeal Surface Electrode. A Method to Reduce the Risk of Recurrent Laryngeal Nerve Injury During Thyroid Surgery. *Langenbecks Arch Surg* (1999) 384(4):392-5. Epub 1999/09/04. doi: 10.1007/s004230050219.

43. Jatzko GR, Lisborg PH, Müller MG, Wette VM. Recurrent Nerve Palsy after Thyroid Operations--Principal Nerve Identification and a Literature Review. *Surgery* (1994) 115(2):139-44. Epub 1994/02/01.

44. Jonas J, Bähr R. [Intraoperative Neuromonitoring of the Recurrent Laryngeal Nerve - Results and Learning Curve]. *Zentralbl Chir* (2006) 131(6):443-8. Epub 2007/01/09. doi: 10.1055/s-2006-955453.

45. Kai H, Xixia L, Miaoyun L, Qinchang C, Xinzhi P, Dingyuan L, et al. Intraoperative Nerve Monitoring Reduces Recurrent Laryngeal Nerve Injury in Geriatric Patients Undergoing Thyroid Surgery. *Acta Otolaryngol* (2017) 137(12):1275-80. Epub 2017/07/26. doi: 10.1080/00016489.2017.1354397.

46. Kotan C, Kösem M, Algün E, Ayakta H, Sönmez R, Söylemez O. Influence of the Refinement of Surgical Technique and Surgeon's Experience on the Rate of Complications after Total Thyroidectomy for Benign Thyroid Disease. *Acta Chir Belg* (2003) 103(3):278-81. Epub 2003/08/14. doi: 10.1080/00015458.2003.11679423.

47. Koulouris C, Papavramidis TS, Pliakos I, Michalopoulos N, Polyzonis M, Sapalidis K, et al. Intraoperative Stimulation Neuromonitoring Versus Intraoperative Continuous Electromyographic Neuromonitoring in Total Thyroidectomy: Identifying Laryngeal Complications. *The American Journal of Surgery* (2012) 204(1):49-53. doi: <https://doi.org/10.1016/j.amjsurg.2011.05.011>.

48. Kunath M, Marusch F, Horschig P, Gastinger I. [the Value of Intraoperative Neuromonitoring in Thyroid Surgery--a Prospective Observational Study with 926 Patients]. *Zentralbl Chir* (2003) 128(3):187-90. Epub 2003/04/16. doi: 10.1055/s-2003-38529.

49. Landerholm K, Wasner AM, Järhult J. Incidence and Risk Factors for Injuries to the Recurrent Laryngeal Nerve During Neck Surgery in the Moderate-Volume Setting. *Langenbecks Arch Surg* (2014) 399(4):509-15. Epub 2014/01/10. doi: 10.1007/s00423-013-1154-6.

50. Lo CY, Kwok KF, Yuen PW. A Prospective Evaluation of Recurrent Laryngeal Nerve Paralysis During Thyroidectomy. *Arch Surg* (2000) 135(2):204-7. Epub 2000/02/11. doi: 10.1001/archsurg.135.2.204.

51. Lorenz K, Abuazab M, Sekulla C, Schneider R, Nguyen Thanh P, Dralle H. Results of Intraoperative Neuromonitoring in Thyroid Surgery and Preoperative Vocal Cord Paralysis. *World J Surg* (2014) 38(3):582-91. Epub 2013/12/19. doi: 10.1007/s00268-013-2402-1.

52. Maneeprasopchoke P, Chongkolwatana C, Pongsapich W, Iwata AJ, Kamani D, Randolph GW. Intraoperative Nerve Monitoring in Thyroid Surgery: Analysis of Recurrent Laryngeal Nerve Identification and Operative Time. *Laryngoscope Investig Otolaryngol* (2021) 6(2):354-61. Epub 2021/04/20. doi: 10.1002/lio2.543.

53. Marcus B, Edwards B, Yoo S, Byrne A, Gupta A, Kandrevas J, et al. Recurrent Laryngeal Nerve Monitoring in Thyroid and Parathyroid Surgery: The University of Michigan Experience. *Laryngoscope* (2003) 113(2):356-61. Epub 2003/02/05. doi: 10.1097/00005537-200302000-00028.

54. Melin M, Schwarz K, Lammers BJ, Goretzki PE. Ionm-Guided Goiter Surgery Leading to Two-Stage Thyroidectomy--Indication and Results. *Langenbecks Arch Surg* (2013) 398(3):411-8. Epub 2012/11/28. doi: 10.1007/s00423-012-1032-7.

55. Melin M, Schwarz K, Pearson MD, Lammers BJ, Goretzki PE. Postoperative Vocal Cord Dysfunction Despite Normal Intraoperative Neuromonitoring: An Unexpected Complication with the Risk of Bilateral Palsy. *World J Surg* (2014) 38(10):2597-602. Epub 2014/05/29. doi: 10.1007/s00268-014-2591-2.

56. Münks S. [Prevention of Recurrent Laryngeal Nerve Paralysis by Demonstration of the Nerve During Thyroid Surgery]. *Laryngorhinootologie* (2005) 84(4):261-5. Epub 2005/04/16. doi: 10.1055/s-2004-826073.

57. Netto Ide P, Vartanian JG, Ferraz PR, Salgado P, Azevedo JB, Toledo RN, et al. Vocal Fold Immobility after Thyroidectomy with Intraoperative Recurrent Laryngeal Nerve Monitoring. *Sao Paulo Med J* (2007) 125(3):186-90. Epub 2007/10/10. doi: 10.1590/s1516-31802007000300011.

58. Page C, Cuvelier P, Biet A, Strunski V. Value of Intra-Operative Neuromonitoring of the Recurrent Laryngeal Nerve in Total Thyroidectomy for Benign Goitre. *J Laryngol Otol* (2015) 129(6):553-7. Epub 2015/06/16. doi: 10.1017/s0022215115001152.

59. Pavier Y, Saroul N, Pereira B, Tauveron I, Gilain L, Mom T. Acute Prediction of Laryngeal Outcome During Thyroid Surgery by Electromyographic Laryngeal Monitoring. *Head Neck* (2015) 37(6):835-9. Epub 2014/03/13. doi: 10.1002/hed.23676.

60. Périé S, Aït-Mansour A, Devos M, Sonji G, Baujat B, St Guily JL. Value of Recurrent Laryngeal Nerve Monitoring in the Operative Strategy During Total Thyroidectomy and Parathyroidectomy. *Eur Ann Otorhinolaryngol Head Neck Dis* (2013) 130(3):131-6. Epub 2013/02/23. doi: 10.1016/j.anorl.2012.09.007.

61. Petro ML, Schweinfurth JM, Petro AB. Transcricothyroid, Intraoperative Monitoring of the Vagus Nerve. *Arch Otolaryngol Head Neck Surg* (2006) 132(6):624-8. Epub 2006/06/21. doi: 10.1001/archotol.132.6.624.

62. Prim MP, de Diego JI, Hardisson D, Madero R, Gavilan J. Factors Related to Nerve Injury and Hypocalcemia in Thyroid Gland Surgery. *Otolaryngol Head Neck Surg* (2001) 124(1):111-4. Epub 2001/03/03. doi: 10.1067/mhn.2001.112305.

63. Robertson ML, Steward DL, Gluckman JL, Welge J. Continuous Laryngeal Nerve Integrity Monitoring During Thyroidectomy: Does It Reduce Risk of Injury? *Otolaryngol Head Neck Surg* (2004) 131(5):596-600. Epub 2004/11/04. doi: 10.1016/j.otohns.2004.05.030.

64. Rosato L, Avenia N, Bernante P, De Palma M, Gulino G, Nasi PG, et al. Complications of Thyroid Surgery: Analysis of a Multicentric Study on 14,934 Patients Operated on in Italy over 5 Years. *World J Surg* (2004) 28(3):271-6. Epub 2004/02/13. doi: 10.1007/s00268-003-6903-1.

65. Runkel N, Riede E, Mann B, Buhr HJ. Surgical Training and Vocal-Cord Paralysis in Benign Thyroid Disease. *Langenbecks Arch Surg* (1998) 383(3-4):240-2. Epub 1998/10/17. doi: 10.1007/s004230050125.

66. Sadowski SM, Soardo P, Leuchter I, Robert JH, Triponez F. Systematic Use of Recurrent Laryngeal Nerve Neuromonitoring Changes the Operative Strategy in Planned Bilateral Thyroidectomy. *Thyroid* (2013) 23(3):329-33. Epub 2012/12/20. doi: 10.1089/thy.2012.0368.

67. Sanguinetti A, Parmeggiani D, Lucchini R, Monacelli M, Triola R, Avenia S, et al. Intraoperative Recurrent Laryngeal Nerve Monitoring in Thyroid Surgery Evaluation of Its Use in Terms of "Spending Review". *Ann Ital Chir* (2014) 85(5):418-21. Epub 2014/03/13.

68. Sarı S, Erbil Y, Sümer A, Agcaoglu O, Bayraktar A, Issever H, et al. Evaluation of Recurrent Laryngeal Nerve Monitoring in Thyroid Surgery. *Int J Surg* (2010) 8(6):474-8. Epub 2010/07/06. doi: 10.1016/j.ijsu.2010.06.009.

69. Schneider R, Lorenz K, Sekulla C, Machens A, Nguyen-Thanh P, Dralle H. [Surgical Strategy During Intended Total Thyroidectomy after Loss of Emg Signal on the First Side of Resection]. *Chirurg* (2015) 86(2):154-63. Epub 2014/05/16. doi: 10.1007/s00104-014-2751-9.

70. Shindo M, Chheda NN. Incidence of Vocal Cord Paralysis with and without Recurrent Laryngeal Nerve Monitoring During Thyroidectomy. *Arch Otolaryngol Head Neck Surg* (2007) 133(5):481-5. Epub 2007/05/25. doi: 10.1001/archotol.133.5.481.

71. Shindo ML, Wu JC, Park EE. Surgical Anatomy of the Recurrent Laryngeal Nerve Revisited. *Otolaryngol Head Neck Surg* (2005) 133(4):514-9. Epub 2005/10/11. doi: 10.1016/j.otohns.2005.07.010.

72. Sitges-Serra A, Fontané J, Dueñas JP, Duque CS, Lorente L, Trillo L, et al. Prospective Study on Loss of Signal on the First Side During Neuromonitoring of the Recurrent Laryngeal Nerve in Total Thyroidectomy. *Br J Surg* (2013) 100(5):662-6. Epub 2013/01/24. doi: 10.1002/bjs.9044.

73. Snyder SK, Sigmond BR, Lairmore TC, Govednik-Horny CM, Janicek AK, Jupiter DC. The Long-Term Impact of Routine Intraoperative Nerve Monitoring During Thyroid and Parathyroid Surgery. *Surgery* (2013) 154(4):704-11; discussion 11-3. Epub 2013/09/07. doi: 10.1016/j.surg.2013.06.039.

74. Steinmüller T, Ulrich F, Rayes N, Lang M, Seehofer D, Tullius SG, et al. Operationsverfahren Und Risikofaktoren in Der Therapie Der Benignen Struma Multinodosaein Statistischer Vergleich Der Komplikationshäufigkeit. *Der Chirurg* (2001) 72(12):1453-7. doi: 10.1007/s001040170010.

75. Steurer M, Passler C, Denk DM, Schneider B, Niederle B, Bigenzahn W. Advantages of Recurrent Laryngeal Nerve Identification in Thyroidectomy and Parathyroidectomy and the Importance of Preoperative and Postoperative Laryngoscopic Examination in More Than 1000 Nerves at Risk. *Laryngoscope* (2002) 112(1):124-33. Epub 2002/01/22. doi: 10.1097/00005537-200201000-00022.

76. Stevens K, Stojadinovic A, Helou LB, Solomon NP, Howard RS, Shriver CD, et al. The Impact of Recurrent Laryngeal Neuromonitoring on Multi-Dimensional Voice Outcomes Following Thyroid Surgery. *J Surg Oncol* (2012) 105(1):4-9. Epub 2011/09/02. doi: 10.1002/jso.22063.

77. Sturniolo G, D'Alia C, Tonante A, Gagliano E, Taranto F, Lo Schiavo MG. The Recurrent Laryngeal Nerve Related to Thyroid Surgery. *Am J Surg* (1999) 177(6):485-8. Epub 1999/07/22. doi: 10.1016/s0002-9610(99)00101-4.

78. Teksoz S, Bukey Y, Ozcan M, Arikan AE, Ozyegin A. Is Nerve Monitoring Required in Total Thyroidectomy? Cerrahpasa Experience. *Indian J Surg* (2015) 77(Suppl 2):466-71. Epub 2016/01/06. doi: 10.1007/s12262-013-0877-5.

79. Thomusch O, Sekulla C, Walls G, Machens A, Dralle H. Intraoperative Neuromonitoring of Surgery for Benign Goiter. *Am J Surg* (2002) 183(6):673-8. Epub 2002/07/04. doi: 10.1016/s0002-9610(02)00856-5.

80. Tschopp KP, Gottardo C. Comparison of Various Methods of Electromyographic Monitoring of the Recurrent Laryngeal Nerve in Thyroid Surgery. *Ann Otol Rhinol Laryngol* (2002) 111(9):811-6. Epub 2002/09/26. doi: 10.1177/000348940211100909.

81. Ulmer C, Koch KP, Seimer A, Molnar V, Meyding-Lamadé U, Thon KP, et al. Real-Time Monitoring of the Recurrent Laryngeal Nerve: An Observational Clinical Trial. *Surgery* (2008) 143(3):359-65. Epub 2008/02/23. doi: 10.1016/j.surg.2007.10.007.

82. Vasileiadis I, Karatzas T, Charitoudis G, Karakostas E, Tseleni-Balafouta S, Kouraklis G. Association of Intraoperative Neuromonitoring with Reduced Recurrent Laryngeal Nerve Injury in Patients Undergoing Total Thyroidectomy. *JAMA Otolaryngol Head Neck Surg* (2016) 142(10):994-1001. Epub 2016/10/22. doi: 10.1001/jamaoto.2016.1954.

83. Witt RL. Recurrent Laryngeal Nerve Electrophysiologic Monitoring in Thyroid Surgery: The Standard of Care? *J Voice* (2005) 19(3):497-500. Epub 2005/08/17. doi: 10.1016/j.jvoice.2004.05.001.

84. Zakaria HM, Al Awad NA, Al Kreedes AS, Al-Mulhim AMA, Al-Sharway MA, Hadi MA, et al. Recurrent Laryngeal Nerve Injury in Thyroid Surgery. *Oman Med J* (2011) 26(1):34-8. doi: 10.5001/omj.2011.09.

**Appendix 2.** References of studies excluded during assessment for eligibility (see Figure 1).

1. Alesina PF, Rolfs T, Hommeltenberg S, Hinrichs J, Meier B, Mohmand W, et al. Intraoperative Neuromonitoring Does Not Reduce the Incidence of Recurrent Laryngeal Nerve Palsy in Thyroid Reoperations: Results of a Retrospective Comparative Analysis. *World J Surg* (2012) 36(6):1348-53. Epub 2012/03/14. doi: 10.1007/s00268-012-1548-6.

2. Andreassen UK, Nielsen TR, Thomsen JC, Balle VH, Brown CL. [Is the Use of Surgical Microscope Justified in Thyroid Surgery? 10-Year Experience with Thyroid Surgery Performed by Means of Microsurgical Technique]. *Ugeskr Laeger* (1999) 161(17):2532-6. Epub 1999/05/18.

3. Atallah I, Dupret A, Carpentier AS, Weingertner AS, Volkmar PP, Rodier JF. Role of Intraoperative Neuromonitoring of the Recurrent Laryngeal Nerve in High-Risk Thyroid Surgery. *J Otolaryngol Head Neck Surg* (2009) 38(6):613-8. Epub 2009/12/05.

4. Aytac B, Karamercan A. Recurrent Laryngeal Nerve Injury and Preservation in Thyroidectomy. *Saudi Med J* (2005) 26(11):1746-9. Epub 2005/11/29.

5. Barczynski M, Konturek A, Cichon S. [Value of the Intraoperative Neuromonitoring in Surgery for Thyroid Cancer in Identification and Prognosis of Function of the Recurrent Laryngeal Nerves]. *Endokrynol Pol* (2006) 57(4):343-6. Epub 2006/09/29.

6. Barczyński M, Konturek A, Pragacz K, Papier A, Stopa M, Nowak W. Intraoperative Nerve Monitoring Can Reduce Prevalence of Recurrent Laryngeal Nerve Injury in Thyroid Reoperations: Results of a Retrospective Cohort Study. *World J Surg* (2014) 38(3):599-606. Epub 2013/10/02. doi: 10.1007/s00268-013-2260-x.

7. Caruso G, Benicchi E, Ciuoli C, Passali FM, Passali D. Inflammatory Bowel Disease: An Increased Risk Factor for Recurrent Laryngeal Nerve Palsy in Thyroid Surgery. *Acta Otorhinolaryngol Ital* (2012) 32(2):130-2. Epub 2012/07/07.

8. Chuang Y-C, Huang S-M. Protective Effect of Intraoperative Nerve Monitoring against Recurrent Laryngeal Nerve Injury During Re-Exploration of the Thyroid. *World J Surg Oncol* (2013) 11:94-. doi: 10.1186/1477-7819-11-94.

9. Cirocchi R, Arezzo A, D'Andrea V, Abraha I, Popivanov GI, Avenia N, et al. Intraoperative Neuromonitoring Versus Visual Nerve Identification for Prevention of Recurrent Laryngeal Nerve Injury in Adults Undergoing Thyroid Surgery. *Cochrane Database Syst Rev* (2019) 1(1):Cd012483. Epub 2019/01/20. doi: 10.1002/14651858.CD012483.pub2.

10. Csáky G, Takács I, Veres L, Boland M. [Early Results after Minimal Lobectomy for Benign Nodular Goiter]. *Magy Seb* (2002) 55(4):268-71. Epub 2002/09/19.

11. Dionigi G, Boni L, Rovera F, Bacuzzi A, Dionigi R. Neuromonitoring and Video-Assisted Thyroidectomy: A Prospective, Randomized Case-Control Evaluation. *Surg Endosc* (2009) 23(5):996-1003. Epub 2008/09/23. doi: 10.1007/s00464-008-0098-3.

12. Hei H, Zhai Y, Qin J, Song Y. Intermittent Intraoperative Neural Monitoring Technology in Minimally Invasive Video-Assisted Thyroidectomy: A Preliminary Study. *J Invest Surg* (2016) 29(2):93-7. Epub 2016/02/19. doi: 10.3109/08941939.2015.1073411.

13. Henry BM, Graves MJ, Vikse J, Sanna B, Pękala PA, Walocha JA, et al. The Current State of Intermittent Intraoperative Neural Monitoring for Prevention of Recurrent Laryngeal Nerve Injury During Thyroidectomy: A Prisma-Compliant Systematic Review of Overlapping Meta-Analyses. *Langenbecks Arch Surg* (2017) 402(4):663-73. Epub 2017/04/06. doi: 10.1007/s00423-017-1580-y.

14. Kasemsuwan L, Nubthuenetr S. Recurrent Laryngeal Nerve Paralysis: A Complication of Thyroidectomy. *J Otolaryngol* (1997) 26(6):365-7. Epub 1998/01/24.

15. Khaled AO, Irfan M, Baharudin A, Shahid H. Comparing the Morbidity of External Laryngeal Nerve Injury in Thyroid Surgery with and without Identifying the Nerve Using Intraoperative Neuromonitoring. *Med J Malaysia* (2012) 67(3):289-92. Epub 2012/10/23.

16. Kim DH, Kim SW, Hwang SH. Intraoperative Neural Monitoring for Early Vocal Cord Function Assessment after Thyroid Surgery: A Systematic Review and Meta-Analysis. *World J Surg* (2021) 45(11):3320-7. Epub 2021/07/01. doi: 10.1007/s00268-021-06225-x.

17. Ku D, Hui M, Cheung P, Chow O, Smith M, Riffat F, et al. Meta-Analysis on Continuous Nerve Monitoring in Thyroidectomies. *Head Neck* (2021) 43(12):3966-78. Epub 2021/08/04. doi: 10.1002/hed.26828.

18. Lee HY, Lee JY, Dionigi G, Bae JW, Kim HY. The Efficacy of Intraoperative Neuromonitoring During Robotic Thyroidectomy: A Prospective, Randomized Case-Control Evaluation. *J Laparoendosc Adv Surg Tech A* (2015) 25(11):908-14. Epub 2015/11/18. doi: 10.1089/lap.2014.0544.

19. Lifante JC, McGill J, Murry T, Aviv JE, Inabnet WB, 3rd. A Prospective, Randomized Trial of Nerve Monitoring of the External Branch of the Superior Laryngeal Nerve During Thyroidectomy under Local/Regional Anesthesia and Iv Sedation. *Surgery* (2009) 146(6):1167-73. Epub 2009/12/05. doi: 10.1016/j.surg.2009.09.023.

20. Lv B, Zhang B, Zeng Q-D. Total Endoscopic Thyroidectomy with Intraoperative Laryngeal Nerve Monitoring. *Int J Endocrinol* (2016) 2016:7381792-. Epub 2016/06/20. doi: 10.1155/2016/7381792.

21. Moroni E, Jonas J, Cavallaro A, Sapienza P, M C, Bahr R. [Intraoperative Neuro-Monitoring of the Recurrent Laryngeal Nerve. Experience of 1000 Consecutive Patients]. *G Chir* (2007) 28(1-2):29-34. Epub 2007/02/23.

22. Pardal-Refoyo JL, Cuello-Azcárate JJ, Ochoa-Sangrador C. [Contribution of Neuromonitoring to the Safety of Tracheal Extubation after Total Thyroidectomy. Prospective Study with Needle Electrodes]. *Rev Esp Anestesiol Reanim* (2013) 60(10):563-70. Epub 2013/09/21. doi: 10.1016/j.redar.2013.06.011.

23. Prokopakis E, Kaprana A, Velegrakis S, Panagiotaki I, Chatzakis N, Iro H, et al. Intraoperative Recurrent Laryngeal Nerve Monitoring in Revision Thyroidectomy. *Eur Arch Otorhinolaryngol* (2013) 270(9):2521-4. Epub 2013/02/02. doi: 10.1007/s00405-012-2338-3.

24. Sanabria A, Kowalski LP, Nixon I, Angelos P, Shaha A, Owen RP, et al. Methodological Quality of Systematic Reviews of Intraoperative Neuromonitoring in Thyroidectomy: A Systematic Review. *JAMA Otolaryngol Head Neck Surg* (2019) 145(6):563-73. Epub 2019/04/12. doi: 10.1001/jamaoto.2019.0092.

25. Sun W, Liu J, Zhang H, Zhang P, Wang Z, Dong W, et al. A Meta-Analysis of Intraoperative Neuromonitoring of Recurrent Laryngeal Nerve Palsy During Thyroid Reoperations. *Clin Endocrinol (Oxf)* (2017) 87(5):572-80. Epub 2017/06/07. doi: 10.1111/cen.13379.

26. T Meyer WH, F Hoppe, et al Intraoperative Neuromonitoring of the Recurrent Laryngeal Nerve. *Chirurgische Praxis* (2002) 60:615-22.

27. Terris DJ, Anderson SK, Watts TL, Chin E. Laryngeal Nerve Monitoring and Minimally Invasive Thyroid Surgery: Complementary Technologies. *Arch Otolaryngol Head Neck Surg* (2007) 133(12):1254-7. Epub 2007/12/19. doi: 10.1001/archotol.133.12.1254.

28. Wahl RA, Rimpl I. [Selective (= Morphology and Function Dependent) Surgery of Nodular Struma: Relationship to Risk of Recurrent Laryngeal Nerve Paralysis by Dissection and Manipulation of the Nerve]. *Langenbecks Arch Chir Suppl Kongressbd* (1998) 115:1051-4. Epub 1999/02/05.

29. Xie Q, Wang P, Yan H, Wang Y. Feasibility and Effectiveness of Intraoperative Nerve Monitoring in Total Endoscopic Thyroidectomy for Thyroid Cancer. *J Laparoendosc Adv Surg Tech A* (2016) 26(2):109-15. Epub 2015/12/23. doi: 10.1089/lap.2015.0401.

30. Yarbrough DE, Thompson GB, Kasperbauer JL, Harper CM, Grant CS. Intraoperative Electromyographic Monitoring of the Recurrent Laryngeal Nerve in Reoperative Thyroid and Parathyroid Surgery. *Surgery* (2004) 136(6):1107-15. Epub 2005/01/20. doi: 10.1016/j.surg.2004.06.040.
